# Italian guidelines for the management and treatment of neonatal cholestasis

**DOI:** 10.1186/s13052-015-0178-7

**Published:** 2015-10-01

**Authors:** Carlo Dani, Simone Pratesi, Francesco Raimondi, Costantino Romagnoli, on behalf of the Task Force for Hyperbilirubinemia of the Italian Society of Neonatology

**Affiliations:** Department of Neurosciences, Psychology, Drug Research and Child Health, Careggi University Hospital of Florence, Largo Brambilla 3, Florence, 50141 Italy; Division of Neonatology, Careggi University Hospital of Florence, Florence, Italy; Division of Neonatology, Section of Pediatrics Department of Translational Medical Sciences, Federico II University of Naples, Naples, Italy; Division of Neonatology, Department of Pediatrics, Catholic University of Sacred Heart, Rome, Italy

**Keywords:** Jaundice, Conjugated hyperbilirubinemia, Cholestasis, Infant

## Abstract

Hyperbilirubinemia is a frequent condition affecting newborns during the first two weeks of life and when it lasts more than 14 days it is defined as prolonged jaundice. This condition requires differential diagnosis between the usually benign unconjugated hyperbilirubinemia and the pathological conjugated hyperbilirubinemia, that is mainly due to neonatal cholestasis. It is important that the diagnosis of neonatal cholestasis be well-timed to optimize its management, prevent worsening of the patient’s outcome, and to avoid premature, painful, expensive, and useless tests. Unfortunately, this does not always occur and, therefore, the Task Force on Hyperbilirubinemia of the Italian Society of Neonatology presents these shared Italian guidelines for the management and treatment of neonatal cholestasis whose overall aim is to provide a useful tool for its assessment for neonatologists and family pediatricians.

## Background

Hyperbilirubinemia is a very common condition that can occur in 2.4 to 15 % of newborns during the first two weeks of life [1]. It is commonly due to an increase in unconjugated bilirubin and resolves spontaneously.

Prolonged jaundice is defined as jaundice lasting more than 14 days or recurring after the second week of life [2, 3]. This condition requires careful evaluation to differentiate unconjugated hyperbilirubinemia, that is usually benign [4], from infrequent conjugated hyperbilirubinemia, that is always pathological [3], and is mainly due to neonatal cholestasis [3–5]. Many pathologies can cause neonatal cholestasis and require medical or surgical treatment. However, even when specific treatment is not available or curative, infants who have cholestasis may benefit from early medical management and optimization of nutrition to prevent complications. Therefore, it is important that the diagnosis of neonatal cholestasis be well-timed, both to prevent the worsening of the patient’s outcome due to a delayed diagnosis [6], and to avoid carrying out premature, painful, expensive, and useless tests. Unfortunately, this does not always occur in practice for several reasons, such as the early hospital discharge of newborns without adequate follow-up of bilirubin serum level, the misleading appearance of pigmented stool, and misdiagnosis of breastfed–associated jaundice. In fact, all these conditions are frequently cited as reasons for late referral of infants for evaluation of cholestasis [7–9].

### Focus of guidelines

These recommendations follow the recent publication of the Italian guidelines for the management of hyperbilirubinemia in infants ≥ 35 weeks’ gestational age [10] and represent their completion. Their overall aim is to provide a useful tool for neonatologists and family pediatricians for the management of newborns with cholestasis and to promote the prompt assessment of infants with prolonged jaundice.

### Methods of statement development

Literature searches were last updated in June 2015.

The hierarchy of evidence from the Center for Evidence-Based-Medicine was applied (see Table 1) [11].

**Table 1:** The hierarchy of evidence from the center for evidence-based-medicine

Level	Definition
1a	Systematic review (with homogeneity) of randomized clinical trials (RCTs)
1b	Individual RCT (with narrow confidence interval)
2a	Systematic review (with homogeneity) of cohort studies
2b	Individual cohort study
3a	Systematic review (with homogeneity) of case–control studies
3b	Individual case–control study
4	Case-series (and poor quality cohort and case–control studies)
5	Expert opinion without explicit critical appraisal, or based on physiology, bench research or “first principles”

### Definition and incidence

Neonatal cholestasis results from impaired bile formation by hepatocytes or from the obstruction of bile flow through the intra- or extrahepatic biliary tree leading to the accumulation of biliary substances (bilirubin, bile acids and cholesterol) in the liver, blood and extrahepatic tissues [3, 4]. Although the laboratory methods used need to be taken into account, neonatal cholestasis can be defined as conjugated hyperbilirubinemia that occurs when conjugated bilirubin is higher than 1 mg/dl, if the total serum bilirubin is ≤5 mg/dl, or >20 % of total serum bilirubin when it is >5 mg/dl [3–5].

The incidence of neonatal cholestasis is approximately 1 in 2,500 live births. The largest diagnostic groups are biliary atresia, that comprises approximately one-third of cases; α_1_-antitrypsin deficiency, that is the cause in 5-15 %; inherited forms of cholestasis which occur in 10 % to 20 % of cases; inborn errors of metabolism and congenital infections (including the TORCH infections), that cause respectively 20 % and 5 % of cases; and parenteral nutrition–associated cholestasis that is the commonest cause in preterm infants [3, 5] (Table 2).

**Table 2:** Differential diagnosis of neonatal cholestasis

Infections	Inherited and metabolic disorders
*Viral*: Cytomegalovirus	α1-antitrypsin deficiency
Rubella	Galactosemia
Reovirus3	Glycogen storage disorder type IV
Adenovirus	Fructosemia
Echovirus	Cystic fibrosis
Coxsackie virus	Hemochromatosis
Human herpes virus 6	Tyrosinemia
Varicella zoster	Arginase deficiency
Herpes simplex	Zellweger’s syndrome
Parvovirus	Dubin-Johnson syndrome
Hepatitis B and C	Rotor syndrome
Human immuno-deficiency virus	Hereditary fructosemia
*Bacterial*: sepsis	Niemann Pick disease, type C
Urinary tract infection	Gaucher’s disease
Syphilis	Wolman’s disease
Listeriosis	Bile acid synthetic disorders
Tuberculosis	Progressive familial intrahepatic cholestasis
*Parasitic*: Toxoplasmosis	North American Indian familial cholestasis
Malaria	Aagenaes syndrome
	X-linked adreno-leukodystrophy
Chromosomal disorders	Vascular disorders
Turner’s syndrome	Budd-Chiari syndrome
Trisomy 18	Neonatal asphyxia
Trisomy 21	Multiple haemangiomata
Trisomy 13	Congestive heart failure
Cat-eye syndrome	
Donahue’s syndrome (Leprechauns)	
Bile duct anomalies	Neoplastic disorders
Biliary atresia	Neonatal leukemia
Choledochal cyst	Histiocytosis X
Alagille syndrome	Neuroblastoma
Non-syndromic bile duct paucity	Hepatoblastoma
Inspissated bile syndrome	Erythrophagocytic lymphohistiocytosis
Caroli syndrome	
Choledocholithiasis	
Gall-stones	
Neonatal sclerosing cholangitis	
Spontaneous common bile duct perforation	
Toxicity	Miscellaneous
Parenteral nutrition	Neonatal lupus erythematosus
Fetal alcohol syndrome	’Le foie vide’ (infantile hepatic non-regenerative disorder)
Drugs	Indian childhood cirrosi
	ARC syndrome (Arthrogryposis, renal tubular dysfunction and cholestasis)

### Clinical presentation

The clinical presentation of neonatal cholestasis may vary in relation to its etiology. Nevertheless, the most common findings in an infant who has cholestasis are prolonged jaundice, acholic stools, dark yellow urine, and hepatomegaly. Jaundice may decrease over the first weeks of life as the indirect bilirubin decreases, thus giving a false impression that the jaundice is resolving. The presence of acholic stools is suggestive, but not diagnostic of extrahepatic biliary obstruction, since this can also be present in severe intrahepatic cholestasis. On the other hand, the presence of pigmented stools suggests patency of the extrahepatic biliary tree and generally makes biliary atresia unlikely. However, in the early course of biliary atresia, stools may appear normally or intermittently pigmented and, therefore, it is important that stool color be serially assessed. Dark urine is common as a non-specific indicator of conjugated hyperbilirubinemia, but is not pathognomonic.

Some infants show coagulopathy secondary to vitamin K malabsorption and deficiency, and present with bleeding (i.e.: gastrointestinal blood loss, bleeding from the umbilical stump, intracranial hemorrhage). Coagulopathy may also be caused by liver failure, indicating either a severe metabolic hepatic disorders (as in respiratory chain deficiency disorders) or cirrhosis and severe liver disease (as in neonatal hemochromatosis). Splenomegaly can be observed in about half of cases, particularly in infants who have infections, cirrhosis and portal hypertension, storage diseases, and hemolytic disorder, but the spleen is usually of normal size in the initial course of extrahepatic biliary obstruction. Congenital infections are associated with microcephaly, low birth weight, intra-uterine growth restriction, chorioretinitis, and thrombocytopenia. Dysmorphic facial features can suggest syndromic and chromosomal disorders.

In an acutely ill infant sepsis, shock, heart failure, hypopituitarism, and metabolic disorders such as galactosemia or tyrosinemia should be evaluated promptly. Neurological abnormalities including irritability, lethargy, poor feeding, hypotonia, or seizures can suggest sepsis.

Ocular manifestations can be found in Alagille syndrome, optic nerve hypoplasia in panhypopituitarism, chorioretinitis in congenital infections, cataract in intra-uterine infections or galactosaemia, and ocular coloboma in Cat Eye syndrome. Cardiac evaluation can reveal peripheral pulmonary stenosis or other cardiac anomalies in Alagille syndrome, dextrocardia in biliary atresia, or patent ductus arteriosus or septum defects in congenital infections. A palpable mass in the right upper quadrant may indicate a choledochal cyst.

The infant’s family history may provide important information, such as consanguinity, while the obstetric history may reveal maternal infection (i.e.: TORCH infection, hepatitis B) or cholestasis of pregnancy (which may be associated with progressive familial intra-hepatic cholestasis).

### Diagnosis

The most important initial investigation is the measurement of fractionated serum bilirubin levels. As aforementioned, infants who have cholestasis will have >1 mg/dL conjugated bilirubin when the total bilirubin is <5 mg/dL or >20 % of the total bilirubin level if total bilirubin is >5 mg/dL. Recently, it has been reported that in the first 4 days of life, the cutoff for elevated conjugated bilirubin may be >0.8 mg/dL and from 8 % to 10 % of the total bilirubin [12]. It has also been suggested that in the first 14 days after birth, the cutoff for elevated conjugated bilirubin may be >0.5 mg/dL, and >2 mg/dL for conjugated bilirubin [13].

Other biochemical markers can be increased in infants with cholestasis, but they are neither diagnostic nor prognostic: a serum transaminase (ALT, AST) increase indicates an unspecific hepatocellular injury; elevated levels of alkaline phosphatase can be found in biliary obstruction but also in the course of bone and kidney diseases. γ-Glutamyl transpeptidase (GGT) is an enzyme in the biliary epithelium whose increase is strongly associated with cholestatic disorders such as biliary atresia, α1-antitrypsin deficiency, Alagille syndrome and idiopathic neonatal hepatitis.

Many further biochemical measurements are useful in identifying the etiology of cholestasis and must be decided on the basis of different clinical situations (i.e.: tests for metabolic inherited disorders, bacterial blood culture or serologies, etc.).

### Chest X-ray

This may be useful for assessing the presence of cardiovascular anomalies, which may be associated with biliary atresia, and detecting skeletal abnormalities characteristic of Alagille syndrome.

### Ultrasonography

Abdominal ultrasound is an important tool in the diagnostic work-up of neonatal cholestasis and is the most useful initial imaging study in the evaluation of neonatal cholestasis [14]. It can assess the size and appearance of the liver and gallbladder--including visualization of gallstones and biliary sludging. An ultrasound examination can establish the diagnosis of choledochal cyst or demonstrate a small or absent gallbladder that may suggest (but is not diagnostic) biliary atresia. On the other hand, the presence of a normal gallbladder does not exclude biliary atresia. The finding of the triangular cord sign, an echogenic area at the porta hepatis due to a fibrous tissue cone, is specific for biliary atresia but is not diagnostic [15, 16]. Common bile duct dilation is not seen in biliary atresia and suggests a distal obstruction or a fruste form of choledochal cyst.

Heart ultrasound should be performed when cardiac anomalies are suspected (i.e.: in case of murmur). In fact, up to 24 % of patients with Alagille syndrome and a subset of biliary atresia patients have structural heart disease [5].

### Radionuclide imaging

Hepatobiliary scintigraphy is carried out using Technetium-99 m-labeled immunodiacetic acid (IDA) derivatives (Tc-99 IDA). The best resolution is achieved if the patient is administered a pretreatment with phenobarbitone (5 mg/kg/d) for at least 3 days previously.. Serial images are taken for up to 24 h or until gut activity is visualized. In a healthy infant, the injected radioisotope is taken up by the hepatocytes, secreted into the biliary system, and then excreted into the small intestine within 24 hours. Slow uptake of the injected radioisotope or nonvisualization of the liver with persistence of the cardiac pool suggests hepatocellular dysfunction, whereas nonvisualization of the radioisotope in the small intestine from 4 to 24 hours suggests either bile duct obstruction or the severe inability of the hepatocyte to secrete. The sensitivity of scintigraphy for biliary atresia is relatively high (83 %–100 %); however, its specificity is low (33 %–80 %) [17, 18]. A recent meta-analysis of 81 studies has shown a pooled sensitivity and specificity of 98.7 % and 70.4 %, respectively [19]. However, when an acholic stool has been seen by an experienced observer scintigraphy adds little information. In fact, since hepatobiliary scintigraphy is expensive, time consuming and poorly specific, many centers do not routinely use this test in the evaluation of cholestatic infants because it may delay the diagnostic evaluation without providing definitive diagnostic information [15]. However, others think that it still has a role where the liver biopsy is ambiguous, and in the evaluation of preterm infants [5].

Recently it has been suggested that singlephoton emission computer tomography (SPECT) can improve the specificity (81.1 %) of hepatobiliary scintigraphy allowing better bowel visualization compared to planar images [19]. However, only two studies have evaluated the SPECT method and further studies are needed [20, 21]. Moreover, also the sampling of gastric and duodenal fluids and measurement of their activities seems to increase the accuracy of hepatobiliary scintigraphy. In particular, gastrointestinal and duodenal fluid sampling increases the specificity to 73.2 and 77.1 %, respectively.

### Magnetic resonance cholangiography (MRC)

Magnetic resonance cholangiography (MRC) can be used to assess the biliary tract. Non- visualization of the common bile duct and the presence of a small gallbladder have been noted in biliary atresia. However, insufficient data are available to recommend routinely this modality [22].

### Endoscopic retrograde cholangiography (ERC)

This method can be useful in the evaluation of infants with biliary obstruction and, in experienced hands, might be a useful tool in the evaluation of certain individual cases. However, the need for high technical expertise and general anesthesia for the study limits its feasibility in some neonates. As a result ERC is of limited usefulness for the evaluation of neonatal cholestasis in most centers [15].

### Liver biopsy

Liver biopsy is the single most definitive investigation in the evaluation of neonatal cholestasis. In several single-center studies, a diagnosis of biliary atresia was correctly suggested by liver biopsy histological findings in 90 to 95 % of cases [23]. The diagnostic histologic appearances of biliary atresia include bile duct proliferation, bile plugs in the portal tract bile duct, portal tract edema and fibrosis. In addition to conventional histology, a wide range of complementary techniques are used, when appropriate, to improve diagnostic yield and can provide the diagnosis of other specific conditions, such as α1-antitrypsin deficiency, some metabolic liver diseases, Alagille syndrome, neonatal sclerosing cholangitis, and viral infection (cytomegalovirus or herpes simplex).

### Management of cholestasis

The first objective in the management of infants with cholestasis is the recognition of diseases amenable to specific medical therapy (i.e.: congenital toxoplasmosis, urinary tract infection, galactosemia, tyrosinemia, hypothyroidism) or early surgical intervention (biliary atresia, choledochal cyst). In the remaining pathologies the medical management of cholestasis is aimed mostly at treating the complications of chronic cholestasis, such as fat malabsorption and fat-soluble vitamin deficiencies, pruritus, hypercholesterolemia, cirrhosis, portal hypertension and liver failure, but cannot change the course of the disease.

Ursodeoxycholic acid (UDCA) has been found to have beneficial effects on many forms of cholestasis, and is generally used as first-line therapy for pruritus due to cholestasis, parenteral nutrition-induced cholestasis, biliary atresia after surgical treatment, and α1-antitrypsin deficiency (evidence level 2b). Its mode of action is not completely understood but appears to have two components: (a) substitution in the bile acid pool for more hepatotoxic hydrophilic bile acids, and (b) stimulation of bile flow. The dosage is 20–30 mg/kg/d in three divided doses [24]. The only common side-effect is diarrhea which usually responds to dose reduction. UDCA can be discontinued when cholestasis has resolved.

Rifampicin inhibits bile acid uptake by hepatocytes and induces hepatic microsomal enzymes. The recommended dosage is 10 mg/kg/d. It is indicated in the management of pruritus, but liver function should be monitored due to potential hepatotoxicity (evidence level 4) [2, 4].

Cholestyramine may be useful in resistant pruritus and severe hypercholesterolemia associated with cholestasis (evidence level 4) [2, 4]. It acts by binding intestinal bile acids and cholesterol, thus preventing reabsorption and promoting bile acid synthesis from cholesterol. However, cholestyramine may have side effects such as metabolic acidosis, steatorrhea, and constipation. Doses of 250 mg/kg/d are generally used.

Phenobarbital stimulates bile acid independent flow, enhances bile acid synthesis, induces hepatic microsomal enzymes and, hence, lowers the circulating bile acid levels, but sedation and behavioral side effects limit its use. The dosage is generally 3–10 mg/kg/d (evidence level 4) [2, 4].

### Nutritional management of cholestatic infants

Infants with cholestasis often present steatorrhea and increased energy expenditure. Therefore, caloric intake should be approximately 125 % of the recommended dietary allowance based on ideal body weight [3, 4]. Medium chain triglycerides (MCT) are more readily absorbed than long chain fatty acids and are a better source of fat calories. In fact, MCTs are relatively water soluble, do not require solubilization by bile acid micelles and can be directly absorbed into the portal circulation (evidence level 5).

The intestinal absorption of fat-soluble vitamins (A, D, E and K) requires the presence of bile acids. Doses of at least two to four times the recommended daily allowance are given. Vitamin supplementation should continue at least three months after resolution of jaundice as there is a delay before normal bile flow is established (evidence level 5) (Table 3).

**Table 3:** Fat-soluble vitamin supplementation in the cholestatic infant

Vitamin	Preparation	Dosage	Adverse effects
Vitamin A	Aquasol A	5000-25000 U/day per os	Hepatotoxicity, Hypercalcemia,
Vitamin D	Cholecalciferol	800-5000 U/day PO per os	Hypercalcemia, Nephrocalcinosis
Vitamin E	D-α-tocopheryl polyethylene glycol 1,000 succinate	15-25 U/kg/day PO per os	Potentiation of vitamin K deficiency coagulopathy
Vitamin K	Phytomenadione	2.5–5 mg twice a week to every day per os	

### Neonatal phototherapy in cholestasis

Bronze baby syndrome is the dark gray-brown pigmentation of skin, mucous membrane and urine following phototherapy that occurs in some infants with cholestasis caused by a poorly understood accumulation of the bilirubin photoisomer, and/or porphyrins, and other metabolites, and/or biliverdin [25]. This syndrome generally recovers spontaneously and cholestasis does not contraindicate phototherapy. The American Academy of Pediatrics suggests that the direct (conjugated) serum bilirubin should not be subtracted from the total serum bilirubin concentration in making decisions about exchange transfusions (evidence level 5) [26]. Moreover, it suggests that in infants who develop the bronze baby syndrome, exchange transfusion should be considered when the total serum bilirubin is in the intensive phototherapy range and phototherapy does not promptly lower the total serum bilirubin (evidence level 5) [26]. In fact, it has been reported that bronze baby syndrome may be an additional risk of developing kernicterus (evidence level 4) [27]. However, the paucity of data do not permit firm recommendations on this topic.

## Specific diseases

### Biliary Atresia

Biliary atresia is an idiopathic fibrosing cholangiopathy of unknown etiology that leads to complete obstruction of the extrahepatic bile duct during the first few months after birth, progressive biliary cirrhosis, and eventual death if left untreated. It occurs in 1 in 6,000 to 18,000 live births and accounts for approximately one-third of the cases of neonatal cholestasis [28]. It is the most important differential diagnosis in neonatal cholestasis because it should be recognized and treated before the infant reaches the 60 days of age.

There are two clinical forms of biliary atresia: 80 % of infants have isolated atresia without other congenital malformations and are labeled as having the perinatal or so-called acquired form; they show normal early growth, and develop or have persisting jaundice and acholic stools at approximately 3 to 6 weeks of age [5]. The remaining 20 % of infants who have biliary atresia have congenital malformations, including biliary atresia splenic malformation syndrome or other isolated major congenital malformations (splenic and hepatic vascular anomalies, situs inversus, congenital heart disease, etc.), and are labeled as having the so-called fetal/embryonic form. These infants may appear jaundiced at birth and remain so [4].

Children with biliary atresia are usually born after a normal pregnancy, show normal early growth, generally have prolonged jaundice, and develop acholic stools at approximately 3 to 6 weeks of age [5]. Then they develop failure to thrive, pruritus and coagulopathy, while physical examination indicates hepatomegaly and splenomegaly. Ascites and other features of cirrhosis may be seen late in the disease process [5].

Biochemical findings, abdominal ultrasound, and hepatobiliary scintigraphy can be suggestive (see above) but are not diagnostic. Liver biopsy can provide the diagnosis in about 94 to 97 % of patients. The classic pathologic finding is the presence of bile duct proliferation and bile plugs with expansion of portal tracts. These findings, combined with polymorphonuclear exudate and cholestasis, are highly suggestive. However, liver biopsy can be ambiguous in up to 10 % of cases, particularly when done early in the disease process (less than 6 weeks age), and in these cases it should be repeated [15].

α_1_-Antitrypsin deficiency has similar presentation as biliary atresia and should be ruled out before laparotomy [4], also because this condition can be worsened by portoenterostomy [29].

The standard treatment of biliary atresia is the Kasai hepatic portoenterostomy with intraoperative cholangiogram to confirm the site of the obstruction before surgery. The rate of success in re-establishing bile flow is dependent on the age of the infant when the hepatic portoenterostomy is performed as well as on the experience of the surgeon [30]. There is up to an 80 % success rate if the surgery takes place at less than 30 to 45 days of age; however, fewer than 20 % of patients who undergo hepatic portoenterostomy at older than 90 days achieve bile drainage [31–33]. For these reasons a universal infant stool color card screening program has been developed in some countries, such as Taiwan [34] and Japan [35], because it allows early diagnosis and better prognosis for patients with biliary atresia and, moreover, has been found cost-effective [36].

The success of surgery is shown by the excretion of bile and improvement of jaundice. The most significant predictive factor of long-term prognosis is resolution of jaundice. Patients who remain jaundiced usually die or have liver transplantation by 8 years age. Jaundice-free patients have a 10-year survival of almost 90 %. However, the majority of patients with biliary atresia have progressive disease, with at least 80 % requiring liver transplantation by age 20 years [37]. Of those who survive into the third decade after birth without transplant, almost all have portal hypertension or other complications of cirrhosis.

### Idiopathic neonatal hepatitis

Idiopathic neonatal hepatitis accounts for 25–30 % of cases of neonatal cholestasis. There is no specific etiology, and it represents an exclusion diagnosis confirmed by specific histologic findings from liver biopsy of lobular disarray, focal hepatic necrosis and giant cell transformation with evidence of extramedullary hematopoiesis and relatively normal portal tracts [2, 4].

These patients are more frequently preterm infants and can be affected by intrauterine growth retardation. The development of cholestasis generally occurs some weeks after birth and may be associated with hepatomegaly, a mild increase in transaminases, and normal or low GGT; acholic stools are uncommon and are adverse prognostic features [2, 4].

Management is usually supportive with nutritional support, vitamin supplementation and treatment of complications of cholestasis. Prognosis is good with >90 % clinical and biochemical recovery by 1 year of age, with little risk for chronic liver disease in familial cases. There is an empirical 1 % recurrence in subsequent siblings [2, 4].

### Cholestasis in preterm infants

Cholestasis is common in very preterm infants and its etiology is multifactorial [38]. The immaturity of biliary excretion can be exacerbated by further cholestatic insults such as perinatal hypoxia, poor enteral feedings, parenteral nutrition, drug toxicity, and sepsis. It has been reported that 18 % to 67 % of infants who receive prolonged courses of parental nutrition (longer than 14 days) develop liver injury and cholestasis [39]. Moreover, the occurrence of cholestasis associated with parenteral nutrition is inversely correlated with birth weight and directly with starvation and [40] duration of parenteral nutrition [41].

The soybean-based lipid emulsion for parenteral nutrition has been suggested to have a role in the pathogenesis of cholestasis in preterm infants due to its phytosterol content which is toxic and can lead to decreasing bile secretion [42], and omega-6 fatty acids, that can act as proinflammatory hepatic agents [43]. Cober *et al.* found that the decrease of this intravenous fat emulsion in parenteral nutrition from 3 to 1 g/kg/d two times per week can reduce total serum bilirubin without affecting growth (evidence level 2b) [44]. However, Nehra *et al*. has recently reported that the inclusion of soybean oil-based lipid emulsion at 1 g/kg/d compared with 2–3 g/kg/d does not reduce the incidence of cholestasis (evidence level 2b) [45]. Fish oil-based lipid emulsions are composed of omega-3 fatty acids and seem to be useful in treating neonatal cholestasis. Premkumar *et al.* demonstrated that fish oil-based lipid emulsion monotherapy is effective in promoting recovery from cholestasis in preterm infants (evidence level 2b) [46]. Moreover, fish oil-based lipid emulsion (i.e. Omegaven®) has been provided to hundreds of infants with cholestasis and available data suggest that from 80 to 90 % of them recover with a decrease in their conjugated bilirubin of up to <0.5 mg/dL. This high response rate explains the difficulty in performing randomized controlled studies to compare the effect of soybean-based and fish oil-based lipid emulsions on liver function (evidence level 2b) [43, 46, 47].

A novel lipid emulsion containing a mixture of soybean oil, medium-chain triglycerides, olive oil, and fish oil (SMOFlipid®) with reduced omega-6 fatty acids, increased omega-3 fatty acids, and enriched in vitamin E was found to decrease the gamma-glutamyl transferase (GGT) serum level [48], oxidative stress [49], and retinopathy of prematurity [50] in preterm infants compared with a soybean oil-based emulsion (evidence level 1b). Another observational study reported that the use of SMOFlipid® in parenteral nutrition is also associated with the decrease in bronchopulmonary dysplasia and a trend toward a lower incidence of cholestasis in premature infants [51] (evidence level 2b).

Because fish oil-based lipid emulsions are so beneficial for the treatment of parenteral nutrition-associated cholestasis, it has been suggested that they may have potential in preventing it, but this has not been confirmed [51, 52] (evidence level 2b) and further studies are needed to elucidate this question in preterm infants. Nevertheless, considering the lack of adverse effects of these emulsions in supplemented infants and that both Omegaven® and SMOFlipid® have been found to prevent cholestasis and hepatic steatosis in the preterm animal model [53], we believe that fish oil-based lipid emulsions should be preferred to soybean-based lipid emulsion for parenteral nutrition in preterm infants from the beginning.

Taurine supplementation seems to offer a significant degree of protection against parenteral nutrition-associated cholestasis, particularly in patients with necrotizing enterocolitis or severe prematurity, but this effect has not yet been definitively demonstrated (evidence level 2b) [54].

The management of cholestasis in preterm infants should include the discontinuation of parenteral nutrition as soon as possible and the promotion of enteral feeding (also trophic feeding) that enhances bile flow, gallbladder contraction, and intestinal motility. Treatment with UDCA (see the section Management of cholestasis) may be given although there is no evidence of its efficacy in this specific pathology. High (12.5 mg/kg/dose every 6 hours for 14 days) [55, 56] and intermediate-dose (5 mg/kg/dose every 6 hours for 14 days) oral erythromycin has been found to decrease the incidence of parenteral nutrition-associated cholestasis in preterm infants who fail to establish adequate enteral nutrition due to its prokinetic action (evidence level 1b). A meta-analysis of these two randomized controlled studies confirmed a significant beneficial effect of erythromycin in preventing parenteral nutrition-associated cholestasis [57].

Cholecystokinin has been suggested for preventing cholelithiasis or liver disease in patients receiving parenteral nutrition by stimulating the gallbladder, but it was not found to be effective in preventing parenteral nutrition-associated cholestasis (evidence level 2b) [58].

The prognosis of preterm infants with cholestasis is generally excellent and discontinuation of parenteral nutrition is followed by its progressive disappearance and normalization of its biochemical markers.

**Fig. 1:**
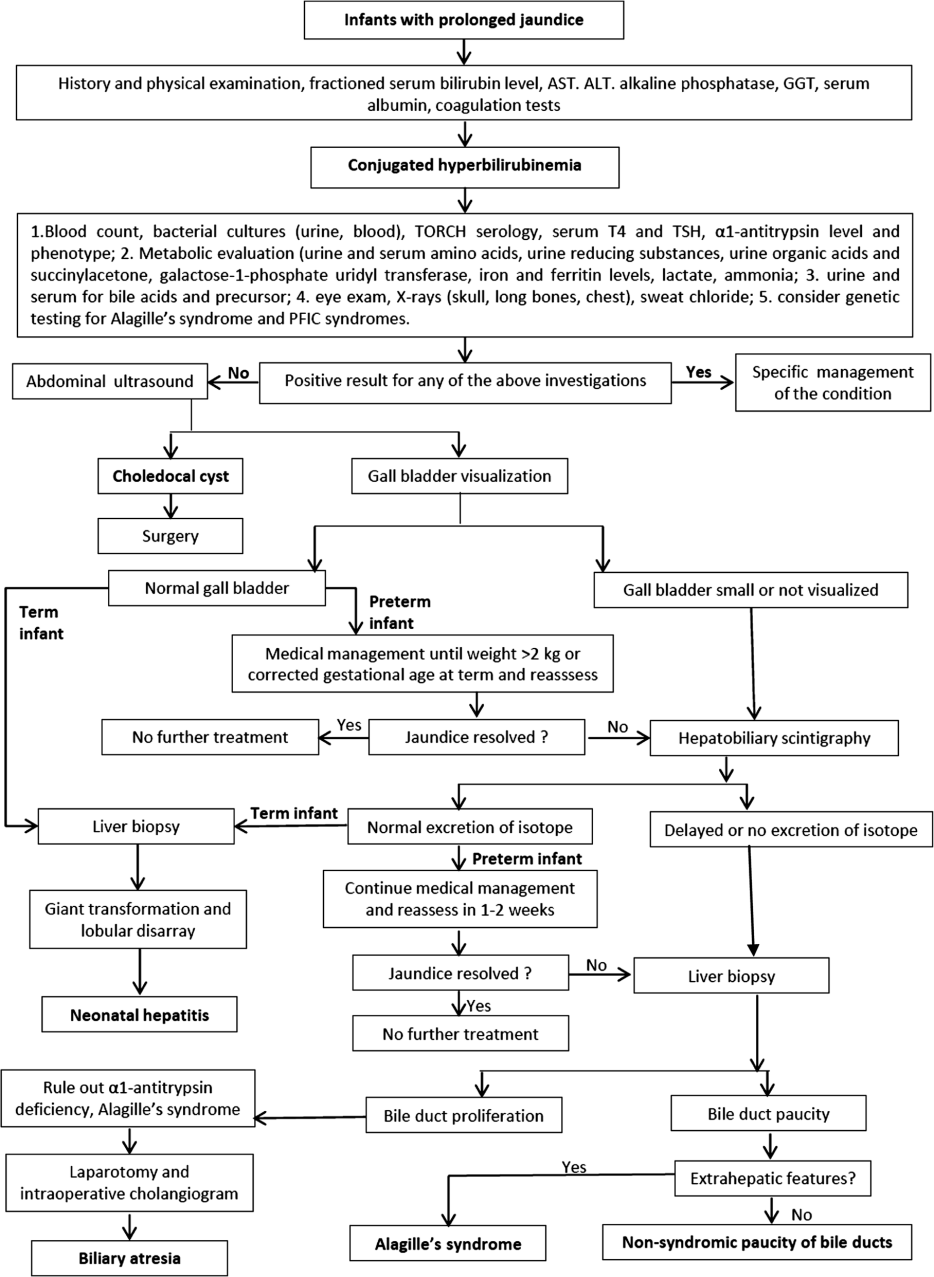
Flow chart for the management of the neonatal cholestasis in term and preterm infants. Modified from ’ with permission

The risk of biliary atresia is uncommon in preterm infants so a modified schedule of investigation is appropriate (Fig. 1). Hepatobiliary scintigraphy and liver biopsy should be delayed until the infant’s corrected gestational age is more than one term and his/her weight is more than 2 kg unless there is biliary dilation. Liver biopsy is indicated in the presence of acholic stools, cholestasis which persists beyond a corrected age of 2–3 months, and in patients who have a nonexcreting hepatobiliary scan [2, 4].

### α1-Antitrypsin deficiency

This autosomal recessive disorder is the most common inherited cause of neonatal cholestasis. α1-Antitrypsin is the most abundant circulating proteinase inhibitor and acts to protect tissues by inhibiting destructive proteases. In patients with α1-antitrypsin deficiency this protein fails to be secreted normally by the hepatocyte, leading to a decrease in its activity in the blood and lungs, and excessive retention in hepatocytes. The mechanism of liver disease is unclear but is related to the accumulation of the mutant **α**1-antitrypsin which can polymerize *in vivo* [59], while its deficiency leads to a failure to neutralize neutrophil lung elastase and causes the early development of emphysema. More than 75 different variants of α1-antitrypsin mutants have been found, M being the normal variant and Z the most common deficient variant [60]. 95 % of the Northern European population are homozygous for the M allele and 2–3 % carry the Z mutation.1:2000–3000 are homozygous for ZZ deficiency which is associated with neonatal liver disease and adult emphysema [61]. However, only 15 % of ZZ neonates develop clinical disease within the first 20 years and the development of disease in individual patients is probably a complex balance between environmental factors which increase **α**1-antitrypsin production, genetic factors which regulate degradation of mutant **α**1-antitrypsin, and physical factors (such as fever) which promote polymerization of the mutant **α**1-antitrypsin [61].

Forty to 50 % of infants who have the ZZ phenotype may have asymptomatic abnormal liver biochemical tests in the first year after birth, and 10 % to 15 % develop neonatal cholestasis. However, fewer than 25 % of those presenting with cholestasis progress to end-stage liver disease during childhood [62], and liver transplantation has resulted in good survival rates of 90 % at 1 year and 80 % at 5 years [63]. Clinical features are similar to biliary atresia but these patients are more likely to have intrauterine growth retardation, and vitamin K-responsive coagulopathy. Diagnosis is made by documenting low plasma levels of α1-antitrypsin and demonstrating the α1-antitrypsin phenotype. Moreover, α1-antitrypsin is an acute phase protein and its value could be normal during inflammation processes, and, therefore, the deficient α1-antitrypsin variants have to be identified by protease inhibitor typing.

There is no specific treatment for α1-antitrypsin deficiency. Management is mostly supportive with nutritional supplementation to maintain adequate growth.

### Progressive familial intrahepatic cholestasis (PFIC)

These are a group of at least three autosomal recessive hereditary disorders characterized by mutations in hepatocellular bile acid transport-system genes leading to impaired bile formation that show progressive intrahepatic cholestasis whose diagnosis must be confirmed by liver histology at biopsy.

In PFIC type 1 and PFIC type 2 patients typically have low or normal GGT levels, low cholesterol levels, and develop early cholestasis. PFIC-1 is caused by mutation in the FIC 1 gene and is the original Byler disease described in the descendants of an Amish American family. It appears to be an allelic disorder with benign recurrent intrahepatic cholestasis. The function of the FIC1 protein that is expressed in hepatocyte canalicular membranes is unknown [64], but patients who have PFIC-1 may also have severe diarrhea, pancreatitis, and hearing loss. Surgical methods such as ileal exclusion and partial external biliary diversion may be effective [65]. Cirrhosis develops by the end of the first decade and liver transplantation is usually required during the second decade.

PFIC-2 is caused by a defect in the canalicular bile salt excretory pump (BSEP). The clinical and biochemical presentation is similar to PFIC-1, but without pancreatitis. Treatment is similar to PFIC-1, but the prognosis is worse, with most patients requiring transplantation in the first decade of life [66]. PFIC-3 is caused by a defect in the canalicular phospholipid transporter MDR3. Clinical presentation is similar to PFIC-1 but is delayed until early adulthood. There may be a history of maternal cholestasis in pregnancy. GGT is markedly elevated and bile analysis shows a high bile acid to phospholipid ratio. Pruritus is less severe than in the other forms of PFIC and is often responsive to UDCA. Treatment is similar to other types, prognosis is variable and difficult to predict in individual cases [64].

### Alagille syndrome

Alagille syndrome is an autosomal dominant multisystem disorder characterized by a paucity of intralobular bile ducts and occurring in approximately 1:70,000-100,000 live births. Almost all patients (90 %) have a mutation in the *JAGGED* 1 gene that encodes a ligand in the Notch signaling pathway, while others have less frequent NOTCH-2 receptor mutations. Patients with Alagille syndrome have a combination of characteristics including chronic cholestasis; a characteristic face with triangular shape, broad forehead, deep-set eyes, small pointed chin, and bulbous nose; skeletal anomalies including butterfly vertebrae, curved phalanges, and short ulna; cardiac anomalies, most commonly peripheral pulmonary stenosis; ocular anomalies such as posterior embryotoxon and optic nerve drusen. Other findings include renal abnormalities such as ectopic kidney; mental retardation and developmental delay; growth retardation, short stature and pancreatic insufficiency.

Infants usually present with neonatal cholestasis. It may be difficult to differentiate from biliary atresia because in some cases initial liver biopsy may show bile duct proliferation, and the characteristic facies may not be evident in the neonatal period. Management is mostly supportive with adequate nutritional intake and treatment of pruritus. Supplementation of fat-soluble vitamins and pancreatic enzymes is needed. The Kasai procedure should be avoided as it does not improve outcome and may worsen it.

The outcome of Alagille syndrome is largely dependent on the individual’s clinical manifestations, especially the severity of the cardiac and renal lesions. For those presenting with cholestatic liver disease in infancy, 20 % to 50 % will require liver transplantation or succumb to cardiac or renal disease by the age of 20 years [67]. More than half of children presenting with neonatal cholestasis progress to cirrhosis and require liver transplantation by age 10. Moreover, the occurrence of hepatocellular carcinoma has been reported in patients with Alagille syndrome [68].

## Recommendations

- Neonates with prolonged jaundice (>14 days) must be assessed for cholestasis by measuring fractioned serum bilirubin level, AST, ALT, alkaline phosphatase, GGT, serum albumin, coagulation tests.
- Neonates with conjugated serum bilirubin >1 mg/dl, if the total serum bilirubin is ≤5 mg/dl, or >20 % of total serum bilirubin, when it is >5 mg/dl, are diagnosed to have neonatal cholestasis.
- Neonates with cholestasis must be checked with biochemical and instrumental tests, and possibly liver biopsy to establish the differential diagnosis as summarized in Fig. 1.
- Late diagnosis of biliary atresia must be avoided because its surgical treatment is much more successful when performed before 30 to 45 days of life.
- The diagnostic approach must be delayed in preterm infants because of the high occurrence of self-limiting parenteral nutrition-associated cholestasis in these patients.
- Fish oil-based lipid emulsions should be used instead of soybean-based lipid emulsion for parenteral nutrition in preterm infants.
- Oral treatment with erythromycin may be considered in preterm infants who fail to establish adequate enteral nutrition due to its prokinetic action for the prevention of parenteral nutrition-associated cholestasis.
- When specific treatments are available they must be started and associated with UCDA treatment at the dosage of 20–30 mg/kg/d in divided doses. UDCA can be discontinued when the cholestasis has resolved.
- Infants with cholestasis must be given a caloric intake of approximately 125 % more than the recommended dietary intake for healthy infants with a preference for MCT as a lipid source. Adequate supplementation with vitamins A, D, E, and K must be guaranteed.
- Cholestasis is not a contraindication for phototherapy. The conjugated serum bilirubin should not be subtracted from the total serum bilirubin concentration in making decisions about exchange transfusions.

## Conclusions

The development of national guidelines helps to standardize the management of neonatal cholestasis both in term and preterm infants. This pathology can be unrecognized or misdiagnosed, leading to a sub-optimal clinical assessment and to a worsening of the patient’s outcome due to a delayed diagnosis. We are confident that these guidelines will enhance the diffusion of a systematic approach to the management of neonatal cholestasis and contribute to limiting the tragic underestimation and late referral of this pathology. The development of a stool color card screening program could be a useful tool for improving the prognosis for patients with biliary atresia in the next future.

## Abbreviations

ALT: Alanine aminotransferase
AST: Aspartate aminotransferase
ERC: Endoscopic retrograde cholangiography
GGT: Gamma-glutamyl transferase
IDA: Immunodiacetic acid
MCT: Medium chain triglycerides
MRC: Magnetic resonance cholangiography
PFIC: Progressive familial intrahepatic cholestasis
SPECT: Singlephoton emission computer tomography
TORCH: Toxoplasma, Rosolia virus, Citomegalovirus, Herpse Simplex virus
UDCA: Ursodeoxycholic acid
